# Altered Markers of Brain Development in Crohn’s Disease with Extraintestinal Manifestations – A Pilot Study

**DOI:** 10.1371/journal.pone.0163202

**Published:** 2016-09-21

**Authors:** Anne K. Thomann, Philipp A. Thomann, Robert C. Wolf, Dusan Hirjak, Christian Schmahl, Matthias P. Ebert, Kristina Szabo, Wolfgang Reindl, Martin Griebe

**Affiliations:** 1 Department of Medicine II, Medical Faculty Mannheim, Heidelberg University, Mannheim, Germany; 2 Department of Psychiatry, Heidelberg University Hospital, Heidelberg, Germany; 3 Department of Psychiatry, Psychotherapy and Psychosomatics, Saarland University, Homburg, Germany; 4 Department of Psychiatry and Psychotherapy, Central Institute of Mental Health, Medical Faculty Mannheim, Heidelberg University, Mannheim, Germany; 5 Department of Psychosomatic Medicine, Central Institute for Mental Health Mannheim, Medical Faculty Mannheim, Heidelberg University, Mannheim, Germany; 6 Department of Neurology, Medical Faculty Mannheim, Heidelberg University, Mannheim, Germany; Universidad de Jaen, SPAIN

## Abstract

**Background and Objective:**

Alterations of brain morphology in Crohn’s disease have been reported, but data is scarce and heterogenous and the possible impact of disease predisposition on brain development is unknown. Assuming a systemic course of the disease, brain involvement seems more probable in presence of extraintestinal manifestations, but this question has not yet been addressed. The present study examined the relationship between Crohn’s disease and brain structure and focused on the connection with extraintestinal manifestations and markers of brain development.

**Methods:**

In a pilot study, brains of 15 patients with Crohn’s disease (of which 9 had a history of extraintestinal manifestations, i.e. arthritis, erythema nodosum and primary sclerosing cholangitis) were compared to matched healthy controls using high resolution magnetic resonance imaging. Patients and controls were tested for depression, fatigue and global cognitive function. Cortical thickness, surface area and folding were determined via cortical surface modeling.

**Results:**

The overall group comparison (i.e. all patients vs. controls) yielded no significant results. In the patient subgroup with extraintestinal manifestations, changes in cortical area and folding, but not thickness, were identified: Patients showed elevated cortical surface area in the left middle frontal lobe (*p*<0.05) and hypergyrification in the left lingual gyrus (*p*<0.001) compared to healthy controls. Hypogyrification of the right insular cortex (*p*<0.05) and hypergyrification of the right anterior cingulate cortex (*p*<0.001) were detected in the subgroup comparison of patients with against without extraintestinal manifestations. *P*-values are corrected for multiple comparisons.

**Conclusions:**

Our findings lend further support to the hypothesis that Crohn’s disease is associated with aberrant brain structure and preliminary support for the hypothesis that these changes are associated with a systemic course of the disease as indicated by extraintestinal manifestations. Changes in cortical surface area and folding suggest a possible involvement of Crohn’s disease or its predisposition during brain development.

## Introduction

Crohn’s disease (CD) is a chronic inflammatory bowel disease (IBD). Its etiopathology is not fully understood, but genetic associations have been found [1–3].

Symptoms include intestinal complaints such as diarrhea and abdominal pains, often accompanied by fever, weight loss and a general malaise. Perianal and intraabdominal fistulas and abscesses as well as intestinal strictures are common complications.

Approximately one third of the patients develop extraintestinal manifestations (EIMs), predominantly affecting the joints, skin, eyes and liver [4–8]. The mechanisms that produce these manifestations are poorly understood, but the occurrence of one EIM seems to predispose patients to others [9], indicating a systemic disease course. Genetic influences also seem to play an important role in the development of EIMs [10, 11].

Neurological and psychiatric phenomena associated with CD have been reported and are associated with a higher risk of cerebrovascular events, polyneuropathy, depression, fatigue and anxiety among others [12–14].

Brain-gut-interactions in general are a topic of growing interest and central mechanisms are thought to influence gut inflammation aswell as symptom generation and processing in IBD and irritable bowel syndrome [15]. The role of brain structure in IBD is still unclear.

Magnetic resonance imaging (MRI) studies have found that patients with CD have reduced gray matter volume and reduced cortical thickness in several cortical regions as well as a higher incidence and number of white matter lesions [16–19].

To our knowledge, no previous studies have specifically examined the brains of patients with a history of EIMs or the possible impact of CD on brain development. Assuming that CD patients with EIMs have a systemic disease that is genetically determined, it seems likely that brain involvement is more common in these patients and that the development of the brain may be affected by the disease. In this early stage of gathering knowledge on brain involvement in CD, it is crucial to understand whether the changes are dynamic and can be seen as a result of inflammation, chronic pain, hypercoagulability and other consequences of the disease or whether they represent a more stable entity that might even precede the „classical”abdominal symptoms of CD.

In this study, we obtained high resolution MRI of 15 patients with CD in remission and 15 matched healthy controls (HC). Applying cortical surface modeling (CSM), we compared cortical thickness, area and folding, the last two measurements representing markers of brain development, and hypothesized that

1. structural changes in CD are more common in patients with a history of EIMs and
2. CD predisposition has an impact on the developing brain.

Our pilot study yielded preliminary support for both hypotheses.

## Materials and Methods

The study was approved by the local ethics committee (Medical Faculty of Mannheim, University of Heidelberg, Germany) in compliance with the Declaration of Helsinki and all participants gave their written informed consent after a thorough explanation of the study design by the investigator.

### Subjects

Fifteen patients with CD from the outpatient clinic for IBD patients at the University Medical Centre Mannheim were evaluated. Data regarding disease duration and course, history of EIM, medication and cardiovascular risk factors (i.e. smoking, hypertension, diabetes or hyperlipidemia) was collected and the patients were asked to complete a patients’ diary for a Crohn’s Disease Activity Index (CDAI) evaluation in the seven days prior to the examination.

The inclusion criteria for patients were as follows: age between 18 and 65 years, duration of Crohn’s disease for more than 2 years, disease in remission for at least six months with a CDAI <150 and righthandedness [20]

Patient-specific exclusion criteria were: active disease as indicated by a CDAI of or above 150, use of steroids or biologics in the last 6 months, currently active EIM, an elevated CRP (>5mg/dl), fever or fecal Calprotectin >150μg/g.

The control group consisted of 15 healthy volunteers matched for age, gender, education, BMI and the aforementioned cardiovascular risk factors.

Individuals with a history of neurological or psychiatric disease, malignancies or with MRI contraindications were excluded from both groups.

All subjects underwent a neurological examination by an experienced neurologist and a neuropsychological screening including an assessment of cognitive function, depression and fatigue symptoms (see below).

The CD group was divided in two subgroups, depending on whether they had a history of EIMs (CD_EIM and CD_noEIM).

### Neurological assessment and cognitive function

A neurological examination was conducted by an experienced resident of the neurological department, including neurocognitive profiling and an extensive physical examination emphasizing screening for potentially vascular or inflammation-mediated symptoms.

Cognitive capacity was evaluated using the Montreal Cognitive Assessment (MoCA), a brief test for the assessment of global cognitive function [21]. It assesses short-term memory, visuospatial function, executive function, attention, concentration and working memory, language, and orientation. Thirty points can be scored, one extra point is given to individuals with less than 13 years of education. The normal score range is from 26 to 30 points.

### Psychological measures

Depression in patients was assessed using the Beck Depression Inventory (BDI)-II [22, 23]. The BDI-II is a 21 item self-report scale with items rated from 0 to 3 points in increasing order of severity. Totaled scores can range from 0 to 63, with higher scores correlating with more severe depression. A total score of 0–13 is considered as minimal, 14–19 is mild, 20–28 is moderate, and 29–63 represents severe depression.

The „Würzburger Erschöpfungsinventar bei Multipler Sklerose/ Wurzburger Fatigue Inventory for MS”(WEIMuS) [24] was originally developed to quantify the degree of physical and cognitive fatigue in patients with multiple sclerosis. It consists of 17 items contributing four points each with the maximum possible score being 68.

A subscore of or above 16 (physical) or 17 (cognitive) or a total score of 32 or more points indicates the presence of fatigue in an individual.

### Image Acquisition

The MRI session was performed on a 3 T MAGNETOM Skyra whole body MR scanner (Siemens Medical Solutions, Erlangen, Germany) using a 20-channel head/neck coil. We used a T1-weighted magnetization prepared rapid gradient echo (MPRAGE) sequence (TR = 1900 ms, TE = 2.13 ms, flip angle = 9°, FoV = 240 × 240, matrix size = 256 × 256, voxel size = 0.9 × 0.9 × 0.9 mm3, slice oversampling = 16.7%, BW = 230 Hz/px, parallel acquisition technique GRAPPA acceleration factor 2).

### Cortical Surface Modeling

Cortical reconstruction was performed with the Freesurfer image analysis suite (Version 5.3.0, http://surfer.nmr.mgh.harvard.edu). The technical details of these procedures are described in previous publications [25–28]. Briefly, this processing includes removal of non-brain tissue using a hybrid watershed/surface deformation procedure [28], automated Talairach transformation, intensity normalization [29], tessellation of the gray matter/ white matter boundary, automated topology correction [30, 31], and surface deformation following intensity gradients for optimal placement of the gray/white and gray/cerebrospinal fluid borders at the location where the greatest shift in intensity defines the transition to the other tissue class [25]. Once the cortical models are complete, a number of deformable procedures can be performed for further data processing and analysis including surface inflation [26], registration to a spherical atlas which utilizes individual cortical folding patterns to match cortical geometry across subjects [27], parcellation of the cerebral cortex into units based on gyral and sulcal structure, and creation of a variety of surface based data including maps of curvature and sulcal depth. This method uses both intensity and continuity information from the entire three dimensional MR volume in segmentation and deformation procedures to produce representations of cortical thickness, calculated as the closest distance from the gray/white boundary to the gray/cerebrospinal fluid boundary at each vertex on the tessellated surface [32]. The maps are created using spatial intensity gradients across tissue classes and are therefore not simply reliant on absolute signal intensity. In addition, the maps produced are not restricted to the voxel resolution of the original data and thus are capable of detecting submillimeter differences between groups. This means that if the pial boundary is between a gray matter and a cerebrospinal fluid voxel, some gray matter change might cause the boundary to move into the voxel [25, 26, 32]. Because of smoothness constraints implemented in Freesurfer, the surface placement and the thickness estimation might reach subvoxel accuracy [25, 26, 32]. This automated approach and the subvoxel accuracy of cortical thickness and cortical area measurements were validated against manual measurements on high-resolution MRI images as well as against neuropathological postmortem analysis [33, 34]: Cortical thickness measurements obtained from these two methods (MRI images vs. neuropathology) agreed within 0.2mm, with a mean difference of 0.077mm [34]. Before statistical analysis, cortical thickness and area maps were smoothed with a 10mm Full Width at Half Maximum (FWHM) Gaussian kernel.

Based on the pial surface reconstruction, an algorithm for measuring 3D local gyrification index (LGI) at each vertex across each hemisphere, including the default smoothing of individual LGI maps, was performed. Details of the LGI computation process can be found in the validation paper [35], previous studies in psychiatric patients [36–38] and at https://surfer.nmr.mgh.harvard.edu/fswiki/LGI. Briefly, an outer envelope that tightly wraps the pial cortical surface is created before local measurement of circular GI is computed for each vertex of the outer surface as the ratio of corresponding regions of interest (ROI) on the outer envelope and pial surface. Delineation of the ROI on both the outer surface (ROI_O_) and pial surface (ROI_P_) uses a matching algorithm based on geodesic constraints, so that the ROI_P_ delineates the entire patch of the cortical surface within the circular perimeter of the ROI_O_. Finally, at the end of the computational process, individual LGI cortical maps quantify the amount of cortex buried within the sulcal folds in the surrounding circular region. Before statistical analysis, individual LGI maps were registered to the fsaverage template included in Freesurfer. As LGI is already smooth, no additional smoothing was applied on the statistical level [39, 40]. The degree of the intrinsic LGI smoothness in our data corresponds to a smoothing kernel of 10 mm.

In Freesurfer, anatomical labeling is based on Talairach coordinates.

### Statistical Analysis

#### Subjects

Sociodemographic and clinical data were evaluated using the Statistical Package of the Social Sciences (IBM SPSS version 23.0). Descriptive and quantitative data were analyzed with Fisher’s exact test, the Mann-Whitney *U*-test or student’s *t*-test, as appropriate.

#### Cortical surface parameters

Using a general linear model (GLM) approach provided by the Query Design Estimate Contrast (QDEC) interface of Freesurfer, we performed a vertex-wise analysis of cortical thickness, area and gyrification over the entire cortical mantle to explore significant differences between groups (i.e. between CD patients and controls, between CD patients with EIM and controls, and between CD patients with and without EIM, respectively).

In order to correct for multiple comparisons, a Monte Carlo simulation with 10.000 iterations and cluster analysis was applied to determine contiguous clusters of significant cortical parameter differences between groups (p < 0.05). The Monte Carlo simulation and clustering approach is implemented in Freesurfer and based on the AlphaSim algorithm (http://afni.nimh.nih.gov/pub/dist/doc/manual/AlphaSim.pdf).

#### Correlation analysis

In order to explore the potential association of morphometric alterations in CD_EIM with duration of the disease, *Pearsons* correlations coefficients were computed in order to examine associations between maxima of cortical alteration and disease duration

## Results

### Sample characteristics (see also S1 Table)

The groups were matched for age, gender, education and cardiovascular risk factors (see Table 1). There were 7 smokers in each group, 3 CD individuals and 4 HC had hypertension (under current treatment), 1 HC had dyslipidemia (high LDL-cholesterol, under current treatment).

**Table 1:** Demographics and clinical characteristics of CD patients and HC.

	CD (n = 15)	HC (n = 15)	*P* value
Gender; male/female	6/9	6/9	1
Age, years; mean (SD)	41.3 (3.5)	41.1 (3.5)	0.979^+^
Education, years; mean (SD)	14.7 (0.7)	15.3 (0.7)	0.478^+^
BMI; mean (SD)	27.1	26.9	0.896^+^
Cardiovascular risk factors; n	9	9	1
-Smokers; n	7	7	1
--packyears; mean (SD)	13,6 (9)	15.3 (12,5)	0.774^+^
-Hypertension; n	4	3	1^+++^
BDI; median (range)	8 (1–17)	4 (0–17)	0.150^++^
WEIMuS; median (range)	13 (0–45)	5 (0–44)	0.160^++^
MoCA; median (range)	28 (26–30)	28 (22–30)	0.846^++^
EIM; n	9	-	-
CDAI; median (range)	26 (0–135)	-	-
Disease duration, years; mean (SD)	18.1 (13.9)	-	-
Fecal Calprotectin, g/kg; median (range)	25,6 (6,9–150)	-	-

BMI, Body mass index; BDI, Beck’s Depression Inventary; CD, Crohn’s Disease; CDAI, CD Activity Index; EIM, extraintestinal manifestations; HC, healthy controls; MoCA, Montreal Cognitive Assessment; SD, standard deviation; WEIMuS, Würzburger Erschöpfungsinventar bei Multipler Sklerose

^+^: student’s t-test;

^++^: Mann-Whitney-U-test,

^+++^: Fisher’s exact test, two-tailed

As summarized in Table 1, there were no significant differences between patients and healthy controls in the psychological and cognitive assessments.

Nine of the 15 individuals with CD reported a history of extraintestinal manifestations (arthritis [n = 4]; erythema nodosum [n = 3], primary sclerosing cholangitis [n = 2]). The CD_EIM subgroup was compared to a matched subgroup of the HC group and to the CD subgroup without EIMs. All subgroups that were compared showed no significant differences in any of the aforementioned variables.

### Cortical Surface Modeling

#### Cortical thickness

No differences in cortical thickness were identified in the group and subgroup comparisons

#### Surface area

In the overall group comparison (i.e. all patients vs. controls), no significant differences were observed.

**Fig 1:**
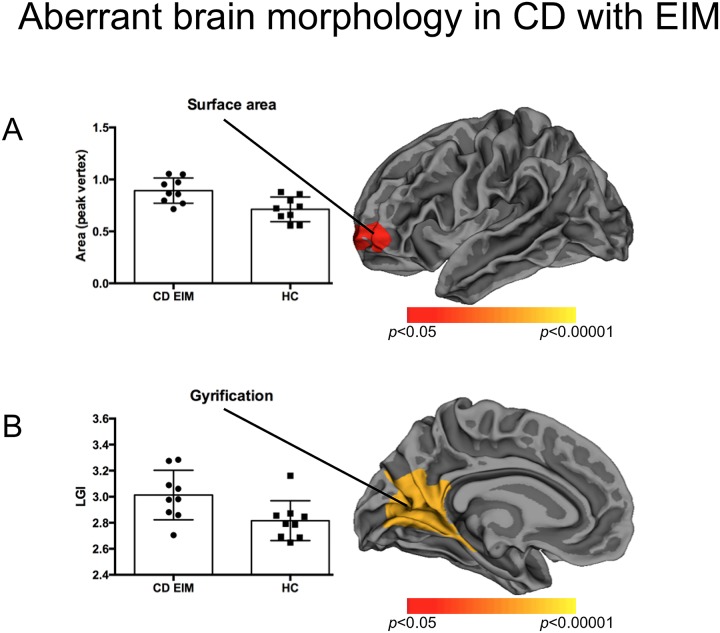
Aberrant brain morphology in CD with EIM. Cortical statistical maps showing left-hemispheric increased surface area (A) and folding (B) in patients with extraintestinal manifestation when compared with controls (p-values corrected for multiple comparisons, vertex-wise analysis over the entire cortical mantle).

In the CD_EIM subgroup, an elevated surface area was found in the left rostral middle frontal gyrus compared to HC (Fig 1A, Table 2, effect size: Cohen’s d = 1,5 (95%CI 0,021–2,979)), whereas the comparison between CD_noEIM and HC yielded no significant differences.

**Table 2:** Brain regions with significant group differences, corrected for multiple comparisons.

Group comparison	Finding	Brain region	Size	Talairach (x y z)	*P*-value*
*CD with EIM vs*. *controls*	Surface area ↑ in patients	Left rostral middle frontal gyrus	1171 mm^2^	-30.8 48.1 -0.2	0.0399
	LGI ↑ in patients	Left lingual gyrus, extending to precuneus, cuneus, PHG and isthmus cingulate	3632 mm^2^	-8.6 -75.9 7.1	0.0001
*CD with EIM vs*. *CD without EIM*	LGI ↑ in CD with EIM	Right rostral ACC, extending to superior frontal gyrus	1980 mm^2^	8.6 33.5 5.3	0.0191
	LGI ↓ in CD with EIM	Right insula	2903 mm^2^	32.1 10.6 10.3	0.0008

ACC, Anterior cingulate cortex; CD, Crohn’s Disease; EIM, Extraintestinal manifestation; GMV, Gray matter volume; LGI, Local gyrification index; PHG, Parahippocampal gyrus

* *P*-values as cluster-wise probability, vertex-wise analysis over the entire cortical mantle

#### Cortical folding

In the overall group comparison (i.e. all patients vs. controls) and the comparison between CD_noEIM and HC, no significant changes were observed.

The CD_EIM subgroup showed elevated LGI in the left lingual gyrus, extending to the left precuneus, cuneus, entorhinal cortex and isthmus cingulate cortex, when compared to HC (Fig 1B, Table 2, effect size: Cohen’s d = 1,141 (95%CI 0,268–2,551)).

**Fig 2:**
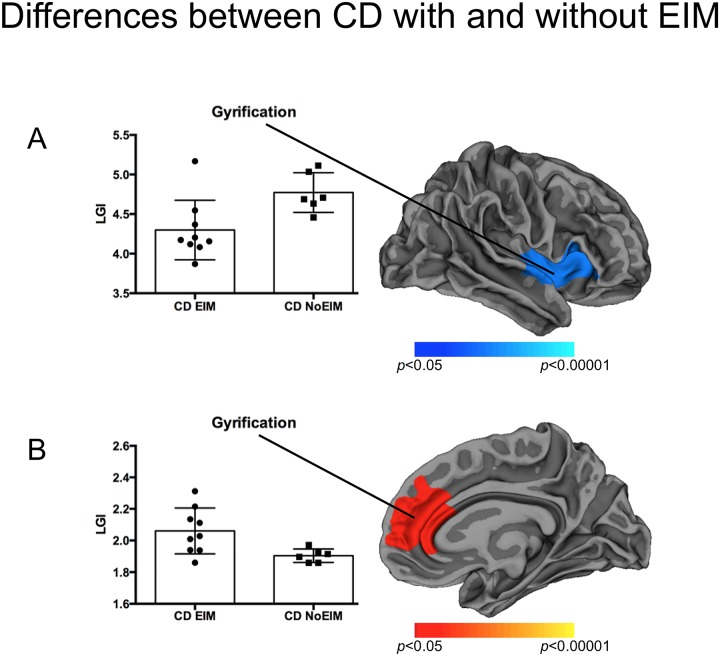
Differences between CD with and without EIM. Cortical statistical maps displaying right-hemispheric lower (A) and higher (B) gyrification in patients with extraintestinal manifestation (p-values corrected for multiple comparisons, vertex-wise analysis over the entire cortical mantle).

When compared to CD_noEIM, a hypogyrification of the right insular cortex, extending to the right orbitofrontal and superior temporal gyrus (Fig 2A, Table 2, effect size: Cohen’s d = 1,421 (95%CI 0,269–2,572)), and a hypergyrification of the right superior frontal gyrus, extending to caudal and rostral anterior cingulate cortex (ACC), were detected in CD_EIM (Fig 2B, Table 2, effect size: Cohen’s d = 1,347 (95%CI 0,207–2,487)).

#### Correlation analysis

None of the observed alterations were significantly correlated with disease duration (r between -0.045 and -0.403; and *p* between 0.282 and 0.90).

## Discussion

The present study investigated the relationship between CD and brain structure applying CSM to analyze high resolution MRI data.

It yielded three major findings:

Firstly, over the whole study sample, i.e. when all CD patients were compared with controls, brain structure as described by cortical thickness, area or folding, did not differ significantly between patients and HC in any brain region. In addition, no correlation of structural changes and clinical variables including disease duration were found.

Secondly, when the CD_EIM subgroup was compared with a matched control group, there were significant differences in cortical area and folding, but no changes in cortical thickness.

The comparison between CD_noEIM and matched HC did not yield any significant differences.

Thirdly, when comparing CD patients with and without previous EIMs, we found significant differences in cortical folding as described by a reduced LGI in the insular cortex and an elevated LGI in the ACC of the CD_EIM subgroup.

Only a few studies using structural MRI-data have investigated the relationship between CD and alterations of brain morphology so far, nearly all of them using voxel-based morphometry (VBM), a well-established method to determine changes in gray and white matter volume. To our knowledge, only one previous study has applied CSM in their analysis [19].

The absence of significant differences in our study when comparing the entire CD group with HC supports the findings of the recent study by Bao et al. using VBM and CSM. The authors described that after correcting for psychological factors (i.e. depressivity and anxiety), the differences in cortical thickness that were first detected were no longer significant. The groups in our study were matched on measures for depression, fatigue and cognitive functioning, which might explain the negative finding.

An earlier VBM study by Agostini and colleagues showed a gray matter decrease in superior and middle frontal areas of both hemispheres and, when applying a ROI-based approach, in the anterior midcingulate cortex [16]. Zikou et al. used VBM in a mixed IBD-group (CD and Ulcerative Colitis (UC)) and found a significant decrease of gray matter volume predominantly in the fusiform gyrus as well as in inferior temporal regions and the right precentral gyrus [18].

The discrepancies in our findings compared with the latter two might at least in part be explained by characteristics of sample selection and imaging modalities, for example patients with UC were explicitly excluded in our analysis. Different approaches of MRI analysis (e.g. VBM vs FreeSurfer) and different MRI-characteristics (e.g. 1.5T vs. 3T) might also explain the difference in findings. The method of VBM yields volume changes in gray and white matter, while CSM determines changes in distinct cortical features that influence gray matter volume. Since cortical volume is a function of both thickness and area [41], it is possible that VBM shows a reduction of gray matter volume while neither thickness nor area alone show significant alterations.

Supplementary, it is worth noting that we saw some trends towards between-group differences with respect to the overall group comparison (i.e. all patients vs. controls), but when corrected for multiple comparisons, none of the effects were significant. However, this might change in a larger sample size.

The sample selection and imaging modalities we applied are best comparable with the study by Bao et al., which supports the compatibility of the findings.

Comparing patients with a history of EIMs against healthy controls as well as against CD patients without EIMs is a novel approach in the observation of the brain’s role in CD.

The evaluation of cortical surface area (CSA) showed a significantly higher value in the CD_EIM group in the rostral middle frontal gyrus. CSA is a variable that varies greatly between individuals and is mainly influenced by genetics [42, 43].

Along with cortical thickness, CSA is directly responsible for total cortical volume, the latter being a product of the former two. However, both are assumed to be phenotypically and genetically independent [41, 44, 45] and to differ in development [46]. According to the radial unit hypothesis, CSA depends on the numbers of cortical columns, while cortical thickness is contingent on the number of neurons in a column [47].

A difference in CSA is thought to originate during brain development rather than from a change of formerly grown substance, although a global decrease is seen in the aging brain [48], probably mirroring reductions in total brain volume [49]. The peak in development of CSA takes place in late childhood [50] and a higher value in local CSA is likely to originate from this lifespan.

We also examined cortical folding. The LGI is a method to evaluate the extent of cortical folding using data processed by the Freesurfer-Software and is used to determine the degree of cortical folding in a brain as described by Zilles et al. [51]. The term LGI is defined as the amount of cortex buried within the sulcus folds compared with the amount of visible cortex in a given area [52]. The development of cortical folding starts prenatally and is mostly completed within the first two years of life, undergoing only minor changes in adolescence [53].

In the CD_EIM subgroup compared to a matched HC-group we found distinctive features in terms of significant hypergyrification in the lingual gyrus, extending to the precuneus, parahippocampal cortex and isthmic cingulate. These regions have been linked to depression in a combined functional and structural MRI study [54].

Comparing the CD_EIM subgroup with the individuals without EIM revealed significant hypergyrification in the ACC and hypogyrification in the insular cortex. Interestingly, both areas also seem to be affected in the aforementioned studies by Agostini et al. and Bao et al. The ACC and the insula are thought to be involved in the emotional processing of grief, sadness and pain [55] and the role of the cingulate cortex in IBD as an important mediator of psychological sympoms and even a potential target for therapy has been discussed by Vogt [56]

The precise relevance and functional meaning of altered gyrification in these areas however remains elusive, as well as of increased CSA in the superior frontal gyrus.

If CSA and cortical folding are more or less fixed in childhood and CD affects their development, a signature of the disease may already be present in the brain before the onset of intestinal and extraintestinal symptoms.

The perinatal phase and early childhood is also critical for the development of the intestinal immune system [57] as the intestine gets colonized after birth, and the early childhood seems to be an important period for the development of a stable intestinal microbiota. An association between gut microbiota and brain development [58, 59] aswell as between gut microbiota and IBD [60–62] have repeatedly been reported. Therefore events during the maturation of the mucosal immune system might also influence brain development.

Previous studies have shown EIMs to be more common in familial IBD [11] and highly concordant in siblings and parent-child-pairs with IBD [10], indicating a genetic association. If EIMs in CD are linked to genetics, this could partly explain that the influence of CD in brain development is stronger in patients who develop EIMs and differs between patients with and without EIMs, as our findings suggest.

We are aware that our findings are preliminary and acknowledge several limitations to this study. The sample sizes, especially of the CD subgroups, are small, and the cross-sectional design doesn’t allow conclusions regarding chronological orders of events. Regarding the psychological scores (BDI and WEIMuS), we saw a trend towards higher values in the CD group, but the differences did not reach statistical significance. This may also be a consequence of the small sample sizes. Larger independent and potentially longitudinal studies are needed to investigate the role of the brain in extraintestinally manifesting CD. However, our results were corrected for multiple comparisons and the effect sizes were large to very large in these small samples, so the approach seems promising for the investigation of larger patient groups.

Future studies should also address the question whether our findings are specific for CD or a common trait in IBD with EIMs; a respective project including patients with UC as well as more CD patients with and without EIMs is currently conducted by our group.

## Conclusion

Our interdisciplinary pilot study gives the first evidence of a relationship between EIMs and altered brain morphology in CD that is independent of psychological factors. The significant alterations of CSA and cortical folding that emerged from this analysis underline the potential relevance of this novel approach with regard to an impact of the disease in brain development.

## Supporting Information

S1 Table Original anonymized data of patients and controls with colorcoded group classification (blue = CD-EIM, orange = CD_noEIM, green = HC matched to CD_EIM) and the respective values for surface area and LGI. (XLSX)
